# Synchronous metastatic omental melanoma and colonic adenocarcinoma: a case report

**DOI:** 10.1186/s13104-015-1099-7

**Published:** 2015-04-03

**Authors:** Constantine Halkias, Jake Sloane, Mohamed Ben-Gashir, Gareth Bashir

**Affiliations:** North Middlesex University Hospital NHS Trust, Sterling Way, London, N18 1QX UK; Division of Surgery and Interventional Science 9th Floor, Royal Free Hospital, University College London, Pond Street, London, NW3 2QG UK

**Keywords:** Omentum, Metastatic, Melanoma

## Abstract

**Background:**

Malignant melanoma is a rare malignancy of the skin with very high mortality rates. Distal metastases are common especially to other areas of the skin, subcutaneous tissues lungs or liver. There are no previously reported cases of skin melanoma metastasizing to the omentum.

**Case presentation:**

A 62 year-old white British man with a past medical history of a malignant melanoma of the skin underwent a laparotomy for a partially obstructing sigmoid tumour. Intra-operatively, a round, smooth textured black lesion was identified on the anterior surface of the omentum; the nodule was confirmed to be a metastatic malignant melanoma with abundant brown pigment and a focal necrotic area.

**Conclusion:**

A metastatic malignant melanoma was discovered incidentally on the omentum during a laparotomy for bowel obstruction. The significance of this is unclear but it is possible that the omentum may have played a protective role in limiting its spread systemically.

## Background

Malignant melanoma, although not the most common malignancy of the skin (less than 5% of skin malignancies), is responsible for over 75 per cent of skin malignancy related deaths [1]. Distant metastases are associated with a 5-year survival of 16% [2]. Common sites for distant metastatic deposits of malignant melanomas are other areas of the skin, subcutaneous tissues, the lungs and the liver.

In this case report we present a patient with an incidental finding of a metastatic melanoma of the omentum, discovered during a laparotomy for bowel obstruction secondary to a primary colonic malignancy.

## Case presentation

A 62 year old white British man presented to our hospital with three day history of left upper quadrant pain, vomiting and abdominal distension. His past medical history included a melanoma in the right side of his face near the angle of his jaw, which was excised in 2006. Physical examination revealed tenderness on the left side of his abdomen. His white blood cell was 15.1 × 10^9/l with a significant neutrophilia of 13.1 × 10^9/l. Other blood investigations were normal. The patient underwent chest and abdominal radiography prior to computer tomography of his abdomen and pelvis.

**Figure 1:**
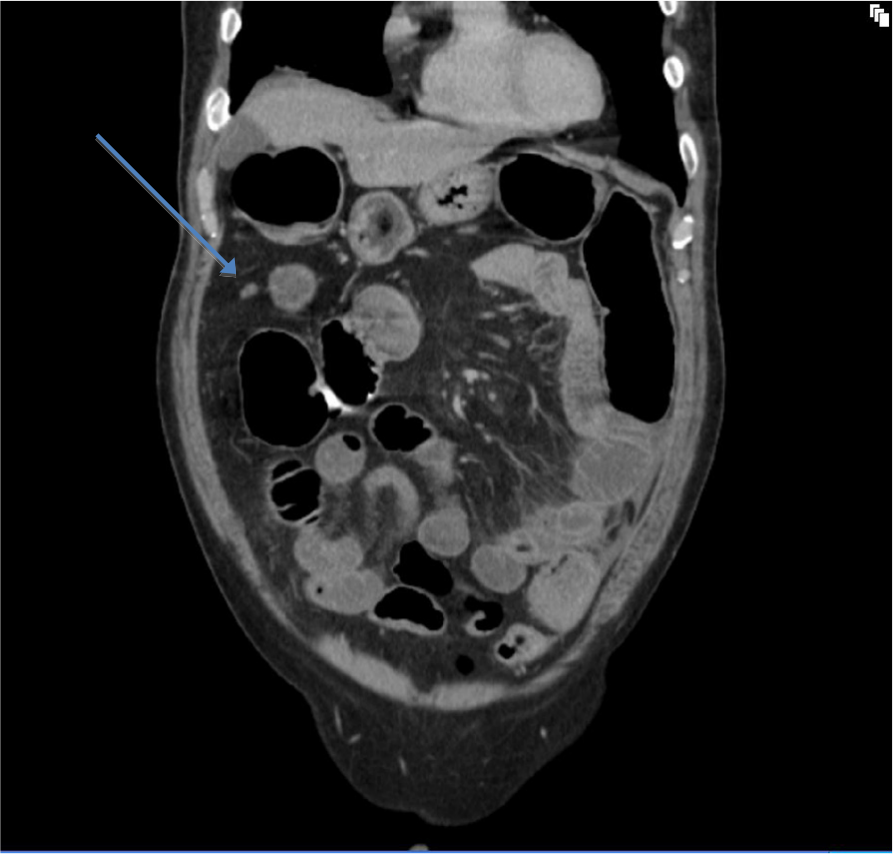
**Coronal section of the CT scan.** The blue arrow is pointing to a small round lesion in the right upper quadrant, most likely representing the omental lesion.

Abdominal radiography revealed distended small bowel loops consistent with large bowel obstruction with an incompetent ileocaecal valve. Computed tomography revealed a lesion in the sigmoid colon with partial obstruction and deposits in the left lung base and peritoneum that were thought to be metastatic in nature (Figure 1).

**Figure 2:**
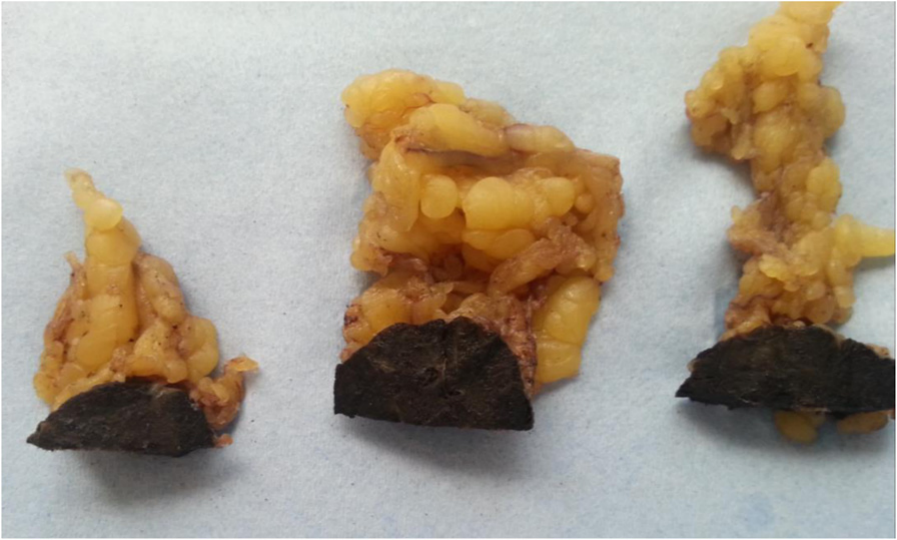
**Macroscopic images of the specimen.**

The patient was resuscitated and operated on the next day. He underwent a left hemicolectomy with a defunctioning ileostomy. Intra-operatively, a round, smooth textured black lesion was identified on the anterior surface of the omentum, measuring 30 × 25 × 22 mm. It was excised along with its surrounding omentum and sent for histopathology separate to the colonic specimen (Figure 2).

**Figure 3:**
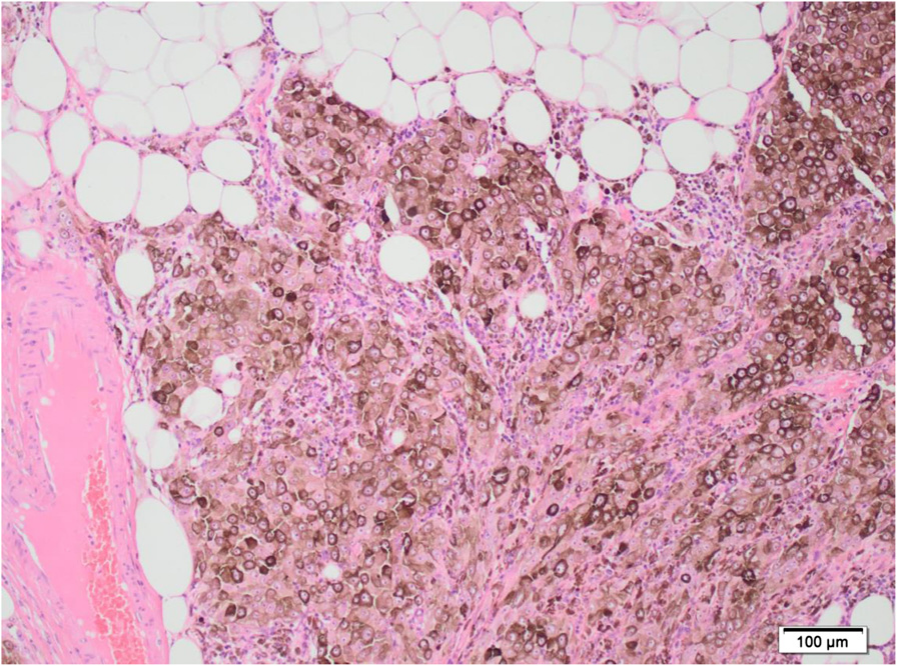
**Microscopic image of the specimen confirming the diagnosis of an omental melanoma.**

The histology report stated a moderately differentiated adenocarcinoma of the left colon with two positive lymph nodes (TNM5 - pT4b, N1, Mx, R0). The omental nodule was confirmed to be a metastatic melanoma (Figure 3).

The metastatic melanoma was 100% clear on H&E sections with nested pattern and abundant brown pigment. Immunohistochemistry confirmed the diagnosis and the melanoma cells were positive for the melanocytic markers S100, MelanA and HMB45.

Post-operative physical examination did not reveal any obvious cutaneous lesions.

## Conclusions

Although visceral and peritoneal metastasis of melanoma is not actually a rare condition, no previous cases of omental metastasis of cutaneous melanoma have been reported. The only similar case, published by *Zhao et al.* [3] is of a melanoma of choroidal origin metastasising to the omentum [3].

Melanoma is an aggressive tumour and metastatic disease has a very poor prognosis.

Resection is the treatment of choice in the absence of disseminated disease [4].

Current guidance [4] in distant metastatic disease, after resection, suggests either a palliative approach or inclusion in a clinical trial.

The mechanisms of metastasis are not very straightforward and a lot is yet to be understood of melanoma’s metastatic behaviour.

There have been several reports of late distant metastases, even 17 [5] and 24 [6] years after the initial diagnosis, and metastases to areas which are not common sites of metastasis [5,7,8]. All the above, are suggestive of a tumour with a behaviour that is not always predictable. In our case it involved the omentum, an extremely rare site for metastasis.

Going through our patient history, the initial cutaneous melanoma excised from the jaw was in fact reported as metastatic in nature (“Dermal deposit of metastatic melanoma”). However, no primary tumour was ever found and hence it was treated as if it were a primary. Given the unpredictable behaviour of these types of tumours, it is difficult to estimate how long the discovered omental lesion had been present. Just as the omentum plays a protective role in isolating sepsis and inflammation in intra-abdominal disease, it is theoretically possible that the omentum may have played a role in containing the melanoma locally.

## Consent

Written informed consent was obtained from the patient for publication of this Case Report and any accompanying images. A copy of the written consent is available for review by the Editor-in-Chief of this journal.

## Abbreviations

TNM: Tumour, Nodes, Metastases
